# Revealing soil legacy phosphorus to promote sustainable agriculture in Brazil

**DOI:** 10.1038/s41598-020-72302-1

**Published:** 2020-09-28

**Authors:** Paulo S. Pavinato, Maurício R. Cherubin, Amin Soltangheisi, Gustavo C. Rocha, Dave R. Chadwick, Davey L. Jones

**Affiliations:** 1 College of Agriculture Luiz de Queiroz - ESALQ-USP, Av. Pádua Dias, 11, Piracicaba-SP, 13418-900 Brazil; 2 School of Natural Sciences, Bangor University, Bangor, LL57 2UW Gwynedd UK

**Keywords:** Pollution remediation, Element cycles

## Abstract

Exploiting native soil phosphorus (P) and the large reservoirs of residual P accumulated over decades of cultivation, namely “legacy P”, has great potential to overcome the high demand of P fertilisers in Brazilian cropping systems. Long-term field experiments have shown that a large proportion (> 70%) of the surplus P added via fertilisers remains in the soil, mainly in forms not readily available to crops. An important issue is if the amount of legacy P mobilized from soil is sufficient for the crop nutritional demand and over how long this stored soil P can be effectively ‘mined’ by crops in a profitable way. Here we mapped the spatial–temporal distribution of legacy P over the past 50 years, and discussed possible agricultural practices that could increase soil legacy P usage by plants in Brazil. Mineral fertiliser and manure applications have resulted in ~ 33.4 Tg of legacy P accumulated in the agricultural soils from 1967 to 2016, with a current annual surplus rate of 1.6 Tg. Following this same rate, soil legacy P may reach up to 106.5 Tg by 2050. Agricultural management practices to enhance soil legacy P usage by crops includes increasing soil pH by liming, crop rotation, double-cropping, inter-season cover crops, no-tillage system and use of modern fertilisers, in addition to more efficient crop varieties and inoculation with P solubilising microorganisms. The adoption of these practices could increase the use efficiency of P, substantially reducing the new input of fertilisers and thus save up to 31.8 Tg of P fertiliser use (US$ 20.8 billion) in the coming decades. Therefore, exploring soil legacy P is imperative to reduce the demand for mineral fertilisers while promoting long-term P sustainability in Brazil.

## Introduction

Achieving food security for a growing global population represents one of the greatest challenges for humankind in the coming decades^1^. The expansion and intensification of existing agricultural lands, especially in tropical areas, stands out as one of the main solutions for increasing food production to meet global demands^2^. Brazil, one of the world's leading producers and suppliers of food, fibres and bioenergy^3^, is an emerging nation whose agriculture has rapidly expanded in recent decades, notably in the Cerrado region (over 204 million hectares (Mha)), and whose land base and deep soils provide large opportunity for conversion of extensive pasturelands into intensive croplands^4^. This land use transition is a promising scenario to allow agricultural expansion in Brazil with minimum environmental impacts^5–7^. However, one major economic and environmental issue associated with expansion and intensification of Brazilian agriculture is the substantial increase in fertiliser demand to sustain crop yields in these new areas (mainly in Cerrado region) characterized by highly-weathered, acid and P-fixing soils^8^.

Phosphorus (P) is an essential element for food and biofuel crop production^9^ and a key nutrient for agriculture expansion in Brazil. Currently, more than 50% of fertiliser P used in Brazilian agriculture is imported^10^, and the internal reserves of phosphate rock, which are of low quality, are estimated to have been used up in around 50 years^11^. Therefore, alternative strategies are needed for Brazilian farming systems to be P sustainable in the future. Exploring the native soil P reservoirs or the residual P that has accumulated over the past 50 years, namely “legacy P”, would facilitate more efficient P use in Brazilian soils. Estimates of global soil P budgets have suggested that most of Brazilian croplands are accruing a P surplus over time^12,13^. This has been confirmed by long-term field experiments, which have shown that a large proportion (> 70%) of the surplus P added to Brazilian soils by fertilisers remains in the soil mainly in forms not readily available to crops^8,14^. These P surpluses represent a legacy P that could be, at least partially, recovered by crops in a profitable way^15,16^.

Legacy P can be found in soils in various chemical species with a continuum of availability, generally classified as readily available, sparingly available and very stable P^17^. The use of soil legacy P by plants is potentially attractive because it provides financial savings on inputs of inorganic P fertilisers, as well as reducing pressure on phosphate rock reserves and reducing the risk of P transfers to water, and hence eutrophication of freshwater and coastal regions. Nevertheless, relevant questions, such as: Is sufficient P mobilized from the soil ‘legacy’ to satisfy crop nutritional needs? and, How long can this stored soil P be effectively ‘mined’ for crop use in a profitable way? still need to be addressed. Central to this is the mapping of legacy P in different soils and agro-climatic regions to allow regionally specific, cost-effective strategies for enhancing crop utilisation of this resource to be developed. Here, we use empirical data to: (i) investigate how agricultural area, P fertiliser consumption and P use efficiency of main crops has evolved over the last 50 years in Brazil; (ii) estimate the total amount of legacy P (agricultural P surplus) accumulated in Brazilian soils over the last 50 years based on P inputs from fertilisers and P outputs by crop harvests, (iii) map the spatial–temporal distribution of soil legacy P across the country; and then (iv) forecast the future agricultural P balance and savings up to 2050, considering potential management strategies to explore more efficiently the use of modern phosphate fertilisers, and soil legacy P for crop production.

## Brazil’s agriculture and reliance on fertiliser P use

**Figure 1:**
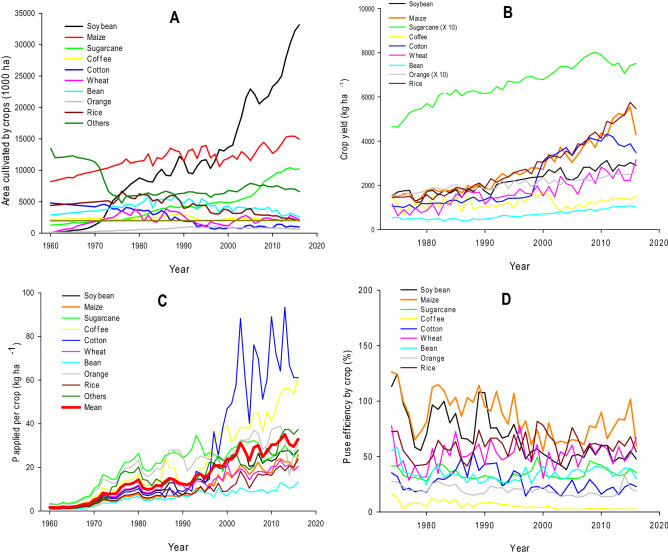
Total cultivated area of the main crops in Brazil (**A**); average crop yield (**B**); average annual application rates of P fertiliser to each crop (**C**); and P use efficiency for each crop (**D**), from 1960 to 2016. Data compiled from Withers et al.^21^, ANDA^10^ and CONAB^41^.

The Brazilian agricultural area extends for over 75.3 Mha^18^, and is currently cropped predominantly with soybean (~ 34.5 Mha) and maize (~ 17 Mha) as annual crops and sugarcane as a semi-perennial crop (~ 9 Mha) (Fig. 1A). Although large, these cropland areas (excluding pastures) comprised less than 9% of the total Brazilian territory in 2016^19^. Since the 1970s, the expansion of cultivation of these three main crops has been substantial (most notably soybean after 2000), representing around 72% of the current cropland area and 90% of the total grain/food/energy production in Brazil^20^. Moreover, sustainable intensification of existing land has been proposed as one of the main strategies to provide global food security^4,20^, although such land intensification is still an enormous political, technological, and social challenge in Brazil.

Natural P scarcity is a major issue in Brazilian soils^21^. The widespread availability and use of P fertilisers, however, has facilitated the transformation of vast unproductive land areas (Cerrado) into profitable agricultural systems. The increase in mineral P fertiliser use over the last five decades has been dramatic, from almost zero in the 1960s to 2.2 Tg P yr^−1^ in 2016^10,22^. The predictions for phosphate mineral fertiliser usage in Brazil is to increase by 3–5% per year over the next decade (~ 3.6% mean increase in Latin America, according to FAO predictions^23^). Further, the amount of P applied per crop has also increased year-on-year in the last two decades (72 and 105% for soybean and maize, respectively), with average values of 27.2 and 22.9 kg ha^−1^ of P applied currently in soybean and maize, the crops responsible for ~ 68% of the cultivated area (Fig. 1C). The amount of P applied to cotton and coffee have also increased, by 137 and 315%, respectively, over the last two decades. However, these two crops represent only ~ 4% of cultivated area, having a small impact on the final quantity of fertiliser applied.

Alongside the expansion of agriculture, Brazilian farmers have changed their soil cultivation system from a predominantly conventional management that included ploughing and harrowing, to a conservation agriculture system (e.g., zero tillage), which represents more than 30 Mha^24^, reducing soil and nutrient losses by erosion and runoff and increasing crop yields^25^. However, despite the increase in P fertiliser usage, P use efficiency (PUE) still remains much lower than expected. In the last decade, PUE has been very low for coffee (~ 2.5%), low for sugarcane, cotton, bean and orange (18–40%), reasonable for soybean, wheat and rice (45–60%) and high only for maize (60–90%) (Fig. 1D). The mean PUE was exactly 50% for the ten main crops from 2000–2016. Lun et al.^13^ have estimated a global mean PUE of 46%, including Brazil with a mean PUE of ~ 60% in croplands, although their estimates were based on a broad-scale view including many generalized assumptions. It is well established that the low PUE values are associated with the high P fixation capacity of Brazilian soils and their ability to quasi-irreversibly bind P on the surfaces of Fe/Al oxyhydroxides^14,22,26^. In contrast to Brazil, temperate countries have a higher average PUE value of 57%^27^, however, this average is still poor when the potential environmental damage arising from excessive P losses is considered. This general inefficiency of P use has created a paradox: how can we increase PUE in tropical soils, like South America and Africa, and how can we avoid P losses via runoff and leaching in regions like Europe, Asia and the USA? According to Jarvie et al.^9^, it is globally imperative to manage both sides of this P paradox to ensure water, energy, and food security for the next generations.

P use efficiency can be increased up to 80% in tropical soils when soil pH is corrected by frequent liming^28^, and crop rotations are adequately used, e.g. well managed long-term soybean/maize rotations intercropped by cover crops under no-tillage cultivation^22^. Such expectations are also supported by Bouwman et al.^29^ who estimated that PUE could reach as high as 64% in Central and Southern America by 2050 just by improving soil and crop management. As a step towards improving PUE, Withers et al.^30^ proposed a 5R stewardship strategy (Re-align P inputs, Reduce P losses, Recycle P in bio-resources, Recover P in wastes, and Redefine P in food systems), which includes many options for more sustainable P use. For example, recently Soltangheisi et al.^31^ estimated that P inputs for sugarcane production in Brazil could be reduced by 63% by 2050 and consequently, the adoption of 5R options would save the sugarcane industry up to US$ 528 million. However, multiple benefits of implementing the 5R strategy for food system resilience and sustainability is dependent on biophysical, socio-economical and institutional involvements^32,33^.

Here we assumed that the average rate of cropland expansion over the last 20 years in Brazil was 2.6% yr^−1^, according to the data presented on Fig. 1A, and that the mean yield of the main crops increased by 58% in the same period, with explicit increases of cotton (152%), rice (108%), maize (90%), bean (66%), wheat (49%) and soybean (27%) (Fig. 1B). The increase in P fertiliser usage over the same period was 5.5% per year^22^. Moreover, areas under double cropping have increased from 3 Mha to nearly 12 Mha over the last 10 years^18^. However, P fertiliser usage under double cropping is proportionally lower than single crop, helping to improve PUE^8^. In addition, according to the predictions of the Brazilian Ministry of Agriculture^34^, it is possible to expand over 70 Mha of new agricultural areas without forest conversion or any other legal restriction^35^. This increase is directly related to the Brazilian green revolution that took place in 1960–1970s^22^. However, it is expected that P fertiliser use per hectare will stabilise in the coming years. It is well known that at the beginning of this historical period of agriculture in Brazil (before 1970s) less P was applied than required by crops. In contrast, current P supply is in excess of crop P requirements. Between 1976–2015, there was an enormous increase in crop yields, from 1.6 to 5.7 t ha^−1^ for maize, 1.3 to 3.1 t ha^−1^ for soybean, 37 to 73 t ha^−1^ for sugarcane^22^, resulting in additional removal of P by these crops. Although P offtake has increased, Roy et al.^8^ have still estimated a current surplus of 14 kg P ha^−1^ yr^−1^ in soybean/maize areas of Mato Grosso State, a representative grain production region of Brazil. It is also supported by Lun et al.^13^ who state that total cropland P inputs of 20 to 25 kg P ha^−1^ yr^−1^ may guarantee high yields while creating a near-equilibrium soil P balance, which is supported by our legacy P data in soybean/maize areas.

In addition to the current large cropping area, 172 Mha of pasture are currently used for extensive grazing by livestock. Most of this pastureland is characterized by low-input systems, chemically poor soils and low stocking rates^20,36^. More recently, with the pressure to expand croplands, pasture reclamation by using new grass varieties and increasing liming and fertiliser use, integrating crop-livestock systems, or changing from pasture to grain/sugarcane crop production are the alternatives for many unprofitable ranchers^4^. However, the use of mineral fertilisers (N-P-K) in pastureland represents only about 1.5% of the current total mineral fertiliser use in Brazil^10^. This scenario could change in the near future once the improvement in the efficiency of pasturelands is mandatory to keep rancher’s profitability and supply the increasing global demand for beef^4^.

## Soil legacy P in Brazilian croplands

Stocks of legacy P are constantly increasing in tropical regions with high P-fixing soils^12,13^, but are spatially heterogeneous at the regional scale and require long-term datasets to be accurately quantified^16,37^. In a global meta-analysis, MacDonald et al.^38^ showed persistent elevation of soil P in cropland across several regions and soil types around the world compared to nearby areas which have never been cultivated. Similar results have also been observed in Brazilian regions^14,22^ with a doubling of total P content in cropland soils compared to native soils. Moreover, P fertiliser is typically applied as soluble inorganic forms, which within the soil profile may be rapidly immobilized by sorption onto soil clay mineral (gibbsite, hematite, goethite) surfaces^17^ or precipitated with Ca, Fe or Al^39^. In this way, soils immobilise this highly labile P and convert it into strongly sorbed moderately and non-labile stable P forms^40^, depending on the intrinsic soil mineralogy. Studies using sequential soil P fractionation schemes and spectroscopic analysis have concluded that legacy P has predominantly accumulated as labile and moderately labile P in temperate soils, and as moderately labile and non-labile P in tropical soils^14,40^. The crop accessibility and successful exploitation of legacy P will consequently depend on its distribution across agricultural soils, soil management, crop rotation (distinct root system), and crop capacity to mobilize the so called ‘non-available’ forms of P.

Here, we estimate the total legacy P in Brazilian soils based on the datasets of cultivated area and yield of the main crops compiled by the 5,563 municipalities from the SIDRA^18^ and CONAB^41^ national database. Annual data of P fertiliser delivery to the farmers by each crop was obtained from ANDA^10^ and the mean P export by each crop was obtained from technical reports and regional references. The overall soil P surplus estimate was based on the crop rotation/succession for annual grain crops (soybean, maize, wheat, cotton and bean) and considered single crops for sugarcane, rice, coffee, orange and others.

**Figure 2:**
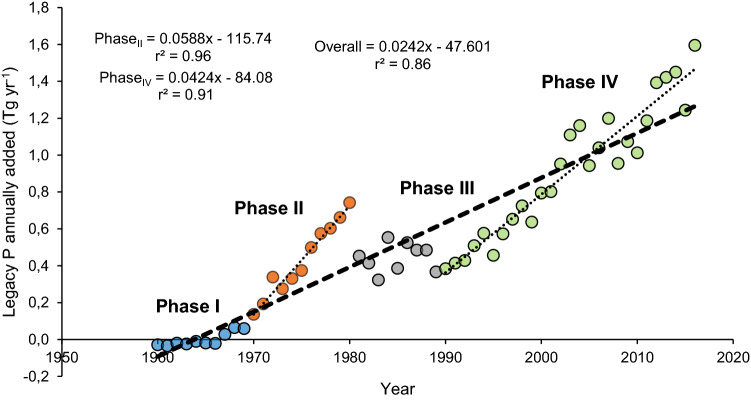
Annual surplus (P input—P output), or legacy P added to the soil over the period 1960–2016 derived from mineral phosphate fertilisers and organic P from manure and industrial by-products used in Brazilian agriculture, considering all crops.

Considering P input from manure plus mineral fertiliser applications to Brazilian soils, and P output data via harvested crops, we calculate a current (2016) annual surplus of 1.6 Tg of P (Fig. 2), which represents ~ 33.4 Tg of legacy P that has accumulated in the soil since 1967 (Supplementary raw data). In monetary value, this represents an estimate of US$ 22 billion accumulated in croplands (considering the current international price of phosphate rock of US$ 86 t^−1^). Although not considered here, before 1967 the P balance in Brazilian soils was negative. Between 1980 and 1990 there was a drop/stability in P accumulation as consequence of the fertiliser price increase (1983) and monetary problems in Brazil during that decade, with a substantial depreciation of farming investment capacity. Between 1967–1980 and 1990–2016, the soil legacy P accumulation increased by 0.059 and 0.042 Tg yr^−1^ of P, respectively. It is estimated that if this pattern seen over the last five decades continues (accumulation rate increasing 0.024 Tg yr^−1^ of P), then the legacy P accumulated by 2050 would reach 106.5 Tg, representing a US$ 70 billion resource residing in the soil, meaning a huge cost for the farming economy and a huge potential environmental risk for surface water contamination (risk of eutrophication by erosion/runoff). Even more alarming is, if the pattern of the last 26 years is considered (Phase IV—Fig. 2), the legacy P is predicted to be 2.84 Tg yr^−1^ by 2050 (total accumulation of 122 Tg), resulting in even more soil residual P accumulation. It is worth noting that all estimations presented here should be interpreted carefully due to potential uncertainties associated with available data and assumptions used for the predicted scenarios and calculations.

**Figure 3:**
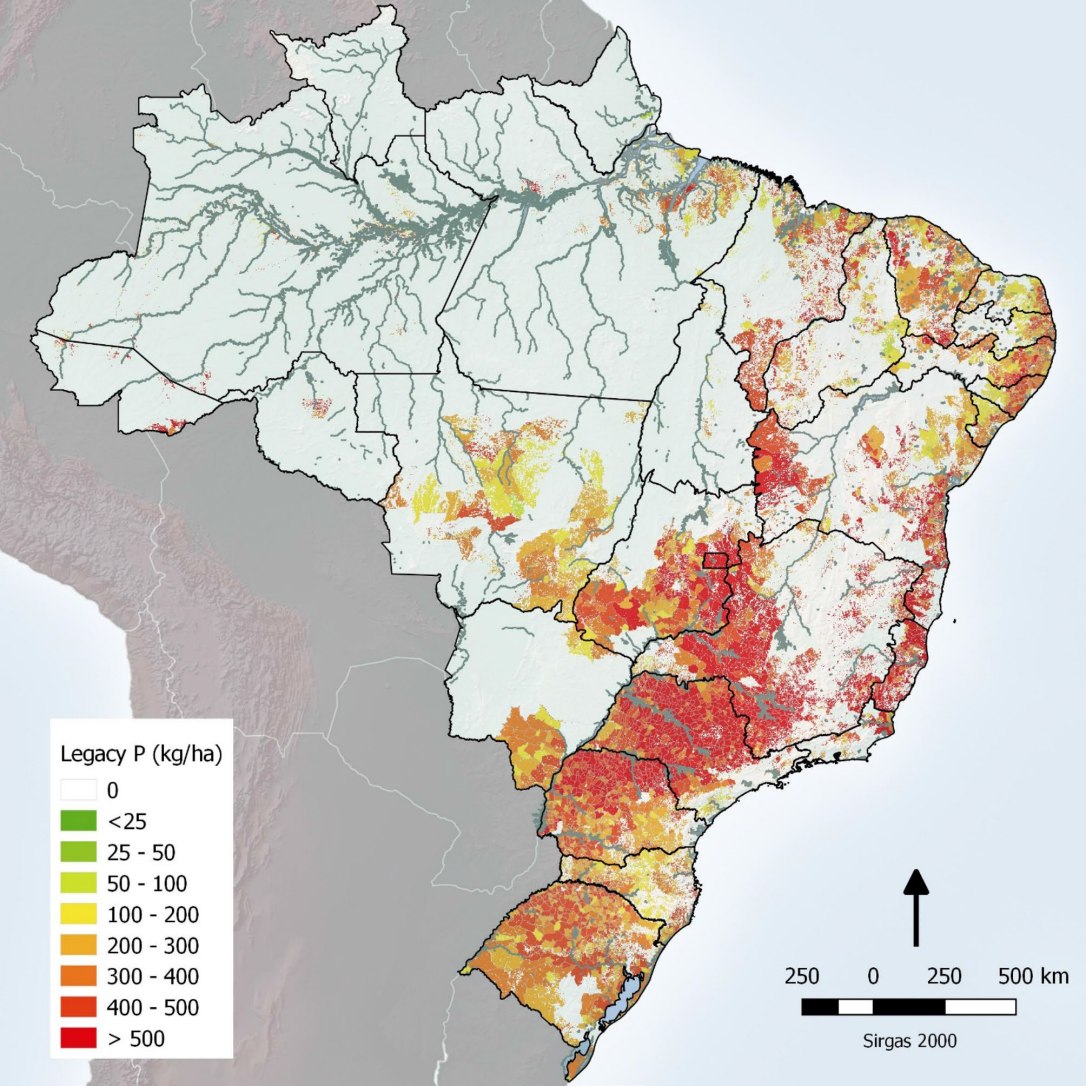
Map of soil legacy P accumulated during cultivation and mineral fertiliser P addition in Brazilian croplands over the period 1960 to 2016. The image comprises all crops and P balances (P input – P output) by municipality (generated by the software QGIS version 3.10—https://qgis.org/en/site/forusers/index.html).

**Figure 4:**
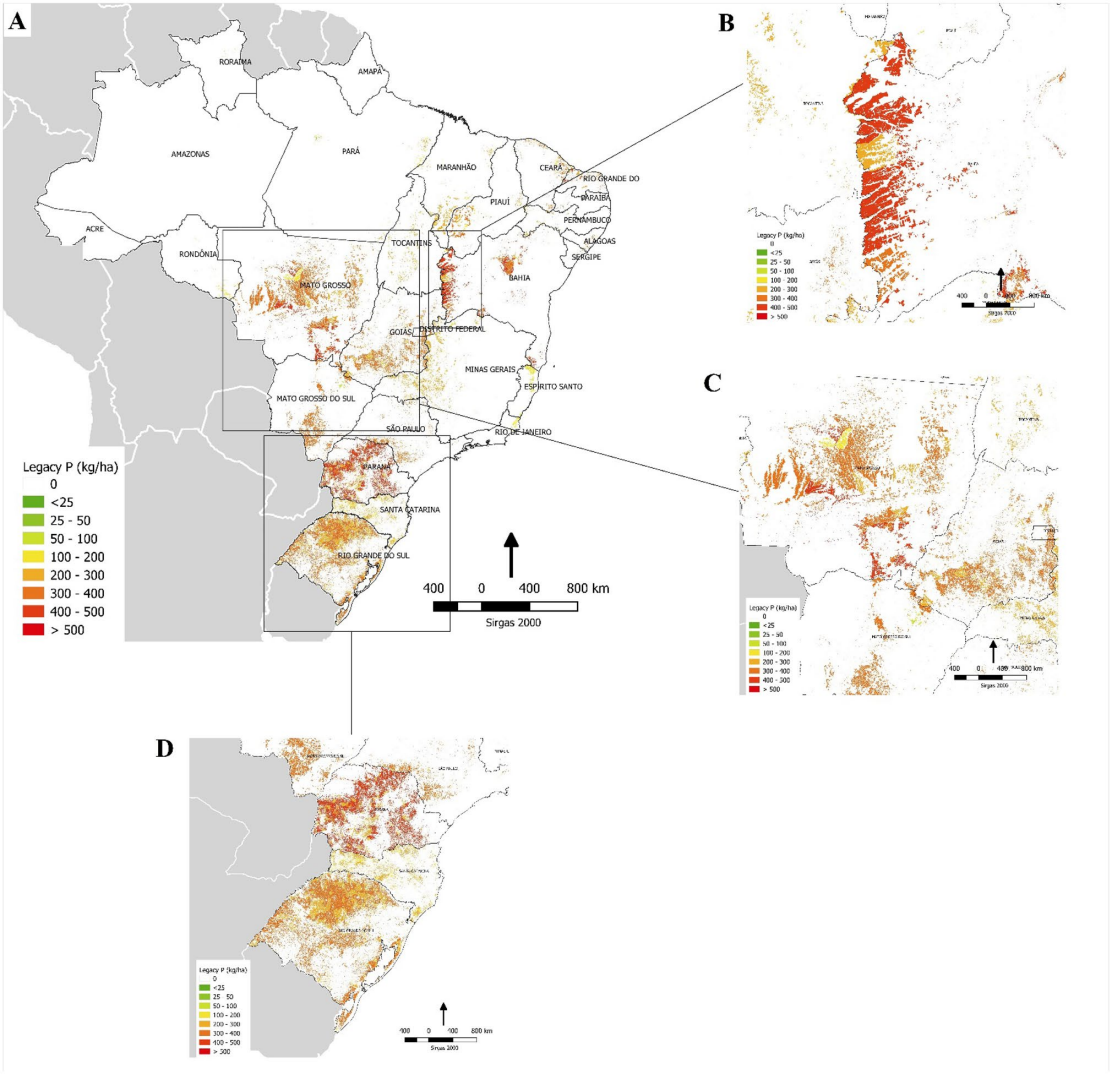
Map of legacy P accumulated from 1960 to 2016 under soybean/maize cultivation in Brazil (**A**), regional accumulation of legacy P under soybean/maize in Northeast – mainly Bahia State (**B**), in Central-West (**C**); and South Brazil (**D**) (generated by the software QGIS version 3.10—https://qgis.org/en/site/forusers/index.html).

The map describing the spatial distribution of the legacy soil P in Brazilian croplands (Fig. 3) and changes in the legacy P distribution through time (Supplementary Figure S2) revealed that higher legacy P values (> 500 kg ha^−1^ of P) are allocated in regions of intensive agriculture in the early stages of the Brazilian green revolution (i.e. early 1970s), used predominantly for sugarcane, orange, coffee and cotton cultivation. In some cases, soil legacy reached up to 1,200 kg ha^−1^ of P in areas constantly cultivated with coffee, the crop with highest demand for P fertiliser and lowest PUE in our estimates. Highest legacy P values were predominantly observed in São Paulo (SP), Minas Gerais and north of Paraná States, as well as some areas in Espírito Santo and Goiás States (Fig. 3). Areas cultivated with soybean and maize still presented a legacy P of between 200–400 kg ha^−1^ (Fig. 4), although these two crops cultivated in rotation or double cropping presented the highest PUE among main crops, with values reaching up to 70–80% when well managed^22^. Soybean and maize cultivation was historically concentrated in the southern regions – especially in Rio Grande do Sul and Paraná States (Fig. 4D); however, they also have followed the agriculture expansion to Cerrado biome in the last decades, including Mato Grosso do Sul, Mato Grosso, Goiás, Bahia and Maranhão States (Fig. 4B,C). Therefore, considering our estimates for Brazil, the surplus of P from 1967 up to 2016 in soils cultivated with soybean alone should be no more than 314 kg ha^−1^ and no more than 124 kg ha^−1^ for maize alone (Supplementary raw data).

Our findings revealed that soil legacy P quantified in Brazil is lower than results reported for other regions of the world with older legacies of agricultural exploitation. According to Sattari et al.^15^, there was an overall cumulative input of P (fertiliser + manure) in Western Europe of 1,115 kg ha^−1^ in croplands for the period 1965–2007, much greater than the cumulative crop P offtake (350 kg ha^−1^). In the same period, in Asia the cumulative input of P was ~ 700 kg ha^−1^ and ~ 500 kg ha^−1^ in North America, Eastern Europe, and Latin America, with an offtake of ~ 250 kg ha^−1^ for all these regions. Compared to Brazil, China’s demand for cereals/legumes and livestock products will continue to increase beyond 2030, even though its growth is predicted to progressively slow down^42^, and China is an important market for Brazilian agricultural products. China also plays a key role in global sustainable P management; from 1970 to 2010, the total P surplus through mineral P application in Chinese croplands was ~ 56 Tg, which represents more than twice the global fertiliser P production in 2010^15,37^. In our estimate, the P surplus in Brazilian croplands from 1967 to 2016 (33.4 Tg) represented nearly half of the total P fertiliser applied in the same period, and represents an average PUE of approximately 50%, smaller than the estimated PUE of ~ 60% by Lun et al.^13^ for Brazil in the period 2002–2010. The P input via mineral fertiliser to soybean, maize and sugarcane, was 798, 597 and 1,263 kg ha^−1^, respectively, with a corresponding crop P offtake of 484, 473 and 420 kg ha^−1^ for 1967–2016 in Brazil. These results emphasize that soybean and maize are P use efficient crops in Brazilian cropping systems, respectively, presenting 60 and 79% of PUE overall during that period, although PUE has decreased more recently (2000–2016) with more intensive use of fertilisers in these two crops (50 and 72%, respectively).

Considering sugarcane, the third most cultivated crop in Brazil, the inorganic P fertiliser use is typically 50–80 kg P ha^−1^ at crop establishment and averages 35 kg P ha^−1^ yr^−1^ overall^31^. Average P export in harvested sugar stalks is only 11 kg P ha^−1^ per year^43^. The recovery of added P fertiliser (~ 31%) leaves large amounts of crop residues in the soil to build up legacy P. We estimated that the legacy P accumulated in the soil since the crop was first cultivated is now > 500 kg ha^−1^ in most of areas of production (Supplementary Figure S1), and this legacy P could be better utilized to improve the resilience of the sugarcane crop to future P shocks^14,16^. This legacy P is largely located in the Central-South region, mostly in São Paulo State where sugarcane cultivation and fertiliser P inputs are concentrated.

## Management strategies to exploit soil legacy P for agricultural production

According to our predictions, the annual surplus of P in Brazilian croplands (fertiliser + manure) may reach up to 2.66 Tg yr^−1^ by 2050, corresponding to an accumulated legacy P of 106.5 Tg, assuming that the rate of expansion seen over the last 50 years continues (Fig. 2). There is a need adopt improved management strategies to exploit the already accumulated soil legacy P (33.4 Tg of P; Fig. 3) and increase PUE across the Brazilian croplands, if we are to address the challenge of increase global agricultural production while preserving natural resources. Some of most promising strategies includes: (i) increasing soil pH by liming, which increases the hydroxyls (HO^−^) in soil solution and consequently increases P availability by competing for the adsorption functional groups of the solid phase^28,39^; (ii) crop breeding seeking varieties with adaptative mechanisms to access previously unexploitable soil legacy P, such as higher root:shoot ratio, altered root morphology (higher presence of hairs, root radius and cluster formation), exudation of chemical compounds into the rhizosphere, and association of roots with mycorrhiza^16,44^; (iii) crop inoculation with P-solubilizing microorganisms^45,46^; (iv) introduction of P-efficient cover crops in the system^47^, e.g. ruzigrass species (*Urochloa* spp)^48^, which has been widely used in intercropping systems in Brazilian Cerrado region; (v) use a more intensive agriculture, with double-cropping or intercropping systems^8^; (vi) use of modern P fertilisers, tuning of fertiliser technologies to better synchronize them with the understanding of plant nutrition and rhizosphere processes and be specific to crops and agro-ecosystems^49^; (vii) adoption of 4R nutrient stewardship to improve P fertiliser management (Right fertiliser source at the Right rate, at the Right time and in the Right place)^50^; viii) improving soil conditions (chemical, physical and biological) for enhancing root growth to explore and uptake P in a large soil volume^47^; and (ix) application of P fertiliser incorporated and/or closer to the plant root system (seed furrow) to facilitate the plant access and minimise the risk of P loss by erosion/runoff.

If most, or at least some of, these strategies are implemented in Brazilian farming systems in the coming years it will be possible to substantially reduce the new input of fertilisers and save up to 31.8 Tg of P being accumulated in the soil by 2050. This saving of this P resource would equate to over US$ 20.8 billion (at current prices) over the next three decades (US$ 86.00 t^−1^ of RP). This estimate is based on the improvement of PUE to 80% in our cultivation systems, which is perfectly plausible following adoption of improved farm management practices according to Withers et al.^22^.

## Concluding remarks

Legacy P accumulated in Brazilian soils currently accounts for 33.4 Tg, and is distributed fairly evenly throughout Brazil’s cropping areas. This legacy P is usually stored in poorly-available forms in Brazilian soils. Soybean and maize represent 44 and 20% of Brazilian cropland area, respectively, and are characterized here as the most P use efficient crops (50 and 72%, respectively). The soils under these crops have the lowest soil legacy P accumulation overall (< 300 kg ha^−1^), much smaller than observed in developed countries^37^, irrespective of the criticism about the inefficiency of intensifying crop production in poor tropical soils^26^.

Our synthesis brings together important information on spatial–temporal distribution of legacy P over Brazilian croplands, at a much greater level of spatial resolution (by municipality) than previous general estimates^15,37^. This level of spatial information is also relevant in identifying the most susceptible regions which are likely to encounter future environmental problems due to excess P in the soil. These data also serve as a scientific basis for integrated modeling, including information on the physical environment (landform, soil type) and management (liming; cover crop; fertiliser use) to model and predict which areas and over what timeframes these areas may need to be managed to reduce the risk of P losses to water bodies and eventual eutrophication problems. Prediction of the P saturation index of cultivated soils may contribute to the understanding of those risks and should be prioritized in future research. Moreover, we propose that our approach to the mapping of legacy soil P can be used as a model of temporal accumulation for other tropical soil regions (i.e. most of Latin America and Africa), with similar soil types and challenges of low PUE.

## Methods

### Estimate of the legacy P accumulated from fertiliser use in Brazilian soils

The estimate of total legacy P in Brazilian soils was based on the total cultivated area and yield from all agricultural crops obtained from the SIDRA—Sistema IBGE de Recuperação Automática^18^ and Companhia Nacional de Abastecimento^41^ databases. Both are official Brazilian government agencies and are annually updated with the actual data of agriculture-livestock production reported at both the State and municipality level. General estimates of soil P surplus were based on mineral + manure P fertiliser addition to the crop rotation/succession for annual grain crops (soybean, maize, wheat, cotton and bean) and for continuous monocultures of sugarcane, rice, coffee, orange and other crops. Potentially, this approach may have slightly under- or over-estimated the total P added and legacy P accumulated in some locations as only the mean of each crop was considered. However, we note that every estimate has an associated inherent level of uncertainty.

The maps of spatial–temporal distribution of legacy P (Figs. 3, 4, S1 and S2) were constructed considering only the mineral P fertiliser input because it was deemed imprecise to predict how much, when and where animal manure or industrial by-products were distributed over the cropped areas in such a large country like Brazil. It is well known that some regions such as Santa Catarina, Rio Grande do Sul, Paraná and Goiás States have used excessive amounts of pig and poultry manure in croplands, in some cases leading to contamination of surface waters (e.g. Santa Catarina)^51^. However, as the spatial distribution of manure addition is uncertain we omitted this from our legacy P maps. Moreover, we did not include fertiliser P applied to cultivated forest soils in our study; although this only accounted for < 1% of P use before 2000 and increased up to 2.5–3.0% beyond 2005. Nor did we include the amount of P applied to grazed grasslands, however, again this only accounted for < 1% of P use before 1994, 2–3% from 1995–2005 and < 2% after 2006^10^.

We considered that limiting the study to mineral P fertilisers used in cropland areas was the most appropriate approach to obtain more realistic distribution of legacy P in each region/ municipality. Further, the total amount of P applied via manure did not constitute more than 15% of total P input after 2000^22^, and consequently is unlikely to interfere severely in our estimates and spatial–temporal maps, exception should be in south Brazil, where the uncertainty is more relevant without this data^51^. In addition, because most Brazilian soils are largely composed of Ferralsols and Ultisols (highly weathered tropical soils), their capacity to adsorb and retain P on soil mineral surfaces (i.e. Fe and Al (hydr)oxides) is very high^14,39^. Potential fertiliser P loss by runoff and leaching was also omitted from our P balance, although potentially this can occur in highly localised situations^13^. As mentioned by Almagro et al^52^, losses by erosion/runoff are influenced by soil management and rainfall regime and are not easy to predict. In this way, P losses by erosion/runoff were not considered here but are an aspect to consider in future evaluations.

Annual data for cultivated area, crop yield and P exported by each of the main crops was based on information from 1974–2016 for each Brazilian State, including soybean, maize, sugarcane, coffee, cotton, wheat, beans, orange, rice and others (e.g. other grain, fruits and vegetables). Data for cultivated area and crop yield from 1960–1973 were estimated according to the observed trend in the following years for each crop (1974–1995) and compared to other smaller published datasets to confirm our estimates^53–55^.

Annual data of P fertiliser delivered to the farmers by State and for individual crops for the period 1986–2016 were obtained from annual reports of ANDA^10^. Previous years, from 1960–1986, were estimated based on the total amount of NPK fertiliser delivered to farmers by manufacturers/ distributors, obtained in other references^56–58^. The estimate of P fertiliser applied to soil by State and by crop in these previous years was obtained considering the average percentage of the period 1986–1996 (a close period without any substantial increase in any specific crop area, according to Fig. 1A). After 1996, the increase in soybean cultivation area was so large that this could have an influence on our estimates. An exception was also made for the Tocantins (founded in 1988) and Mato Grosso do Sul (founded in 1978) States, whose numbers were estimated according to the States they constituted before (Goiás and Mato Grosso respectively).

Mean P offtake by each crop harvested was obtained from technical reports and references presented in Table 1. Accordingly, the annual total P export by each crop was estimated considering crop yield and mean P offtake. To establish the balance of P in cultivated areas, for sugarcane, coffee, orange, rice and others we assumed continuous cultivation in the same area (although it is known that some minor variation has occurred over our study time in some regions/locations). For soybean, maize, cotton, wheat and beans we assumed that the crop rotation over the years/seasons followed the proportional area of each cultivated crop, varying substantially year-by-year (Supplementary raw data).

**Table 1:** Quantity of P exported in the typical harvest of each crop considered in our estimates for legacy P in Brazilian soils.

Crop	P offtake	Unit	References
Soybean	4.60	kg ton^−1^ grain	Francisco et al.^63^
Maize	3.60	kg ton^−1^ grain	Pauletti^64^; Broch & Ranno^65^; Corrêa et al.^66^
Sugarcane	0.13	kg ton^−1^ stalks	Rossetto et al.^67^; Prado et al.^62^
Coffee	1.00	kg ton^−1^ grain	Malavolta^68^
Cotton	4.00	kg ton^−1^ feather + core	SLC Agricola SA (personal information)
Wheat	4.37	kg ton^−1^ grain	Corrêa et al.^66^
Beans	4.00	kg ton^−1^ grain	Pauletti^64^
Orange	0.22	kg ton^−1^ fruit	Malavolta^68^
Rice	2.36	kg ton^−1^ grain	Corrêa et al.^66^
Others	General mean	kg ton^−1^ product	authors

### Mapping the spatial/temporal distribution of legacy P in Brazilian soils

Once the legacy P data had been compiled for each municipality and crop, the information was transformed into a map for the entire Brazilian territory by year of cultivation. The input of P via fertiliser separated by crop and by municipality, and the average values of legacy P (kg ha^−1^) remaining in the soil was added to each specific pixel. Using the QGIS^59^, the information contained in our spreadsheets were georeferenced and transferred to the vector of Brazilian municipalities^18^.

The study reported by Dias et al.^20^ recreates, in pixels of 1 × 1 km, a probabilistic surface (0–100) of the main land uses in Brazil. This study was the basis for the information obtained by municipality. For the entire Brazilian territory, only areas with a probability of having agriculture greater than 9% were considered by each municipality. Therefore, the average values of legacy P were only transferred to these pixels. This process resulted in continuous legacy P surfaces for the period from 1970 to 2016 (1970, 1975, 1980, 1985, 1990, 1995, 2000, 2005, 2010 and 2016) presented in Supplementary Figure S2.

### Organic legacy P

Although not considered in our spatial distribution map (Fig. 3), the overall return of organic P was included in our estimation presented in Fig. 2. Organic P amendment was estimated according to the livestock production considering here poultry, pigs and confined cattle, and industrial by-products like filter cake (FC). We assumed here that grazing cattle do not provide manure for cultivated croplands. Estimates of P in pig and poultry manure follow the numbers presented by Withers et al.^22^. A slight difference in the estimates of confined cattle manure was included here, since the current trend is to improve more efficient systems for cattle production in Brazil, resulting in a more concentrated generation of animal manure^60,61^. We assumed that cattle deliver ca. 7 kg P year^−1^ per animal unit, this is a mean for beef and dairy cattle^15^. Filter cake produced from the processing of sugarcane was also considered as an important way to recycle P. Here, we estimated the amount of P in FC based on sugarcane production (assuming 100% recovery of FC) and considering 10.5 kg of FC dry mass (DM) per ton of cane processed, with 0.80% of P in FC DM tissue^62^. Biosolids from human wastewater were not considered here since the amount treated and potentially used in agriculture is currently very small, less than 33 Gg of P in 2016, with estimates up to 88 Gg by 2050^22^. Most of biosolids area currently disposed in landfills.

As mentioned before, we noted that the distribution of organic P is not uniform over the cropland, however, there is no precise information about that, although it is mostly concentrated close to the production units. Therefore, we opted not to include it in our legacy P map, despite the knowledge that it represented 0.35 Tg year^−1^ in 2016 and is predicted to reach up to 0.57 Tg year^−1^ by 2050 (Supplementary raw data).

## Supplementary information

Supplementary information

Supplementary file2

Supplementary file3
