# Supplementary figures and images for: Revealing soil legacy phosphorus to promote sustainable agriculture in Brazil
Source: Sci Rep. 2020 Sep 28;10:15615. doi: 10.1038/s41598-020-72302-1 (PMC7522976; doi:10.1038/s41598-020-72302-1)

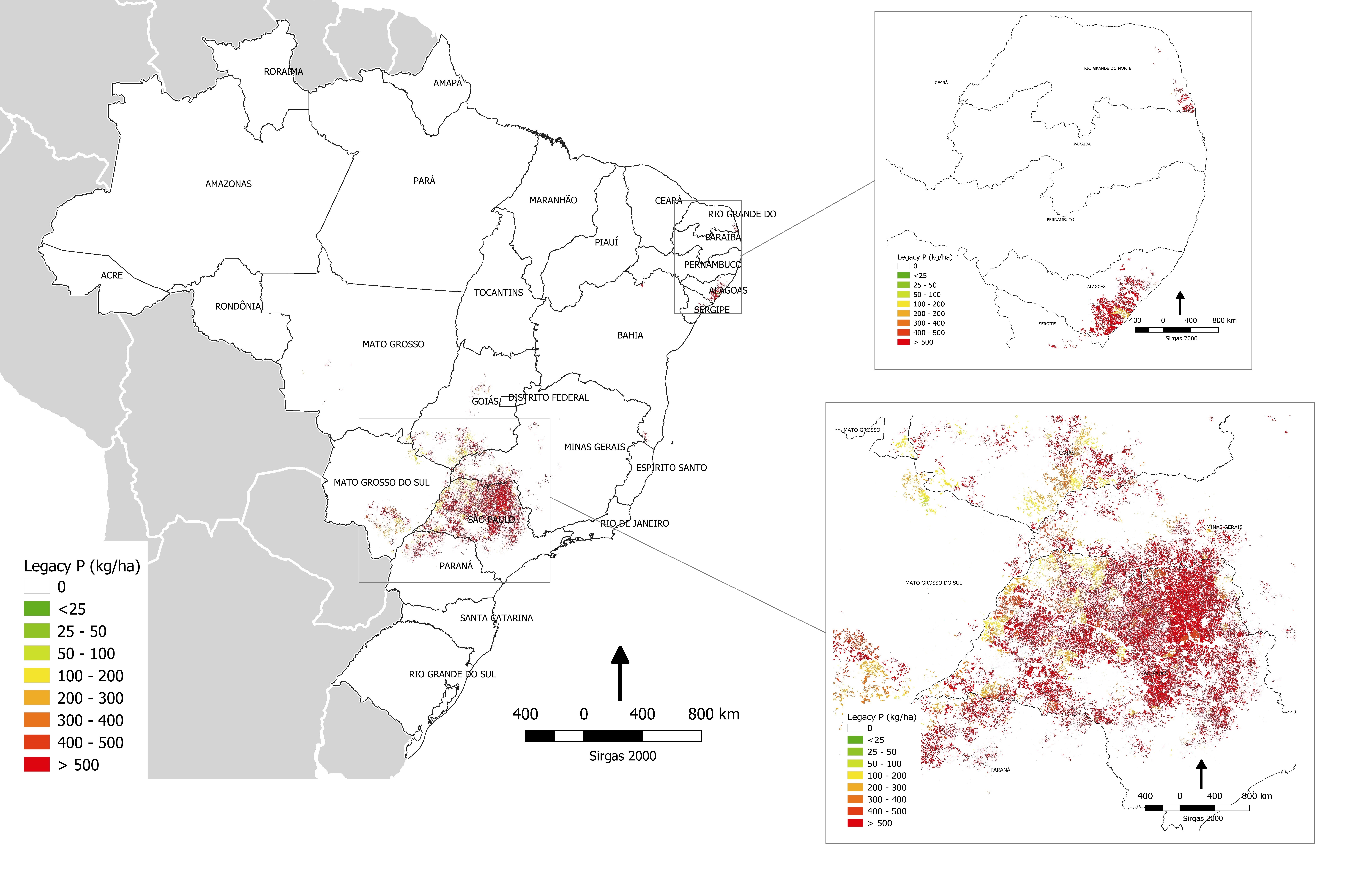

Supplement: Supplementary file 2 — Supplementary file2 [file 41598_2020_72302_MOESM2_ESM.jpg]
